# Everybody's Page

**Published:** 1892-12-10

**Authors:** 


### EVERYBODY'S PAGE.

It would be interesting, though, perhaps, unpleasant, to know how many cemeteries are drained into rivers from which a water supply is drawn. Oxford is stated to have four burial grounds which drain into the Thames, and that city is probably only one amongst many which can boast of an equally sanitary arrangement.

Is it such a very wicked and heartless practice to sell paregoric with opium purposely omitted? A herbalist summoned recently for selling the drug without any opium in it was acquitted because the tribunal and witnesses, as Mr. John Attfield, F.R.S., has pointed out, were under the impression that paregoric is not in the British Pharmacopœia, whereas it has been included since 1888. The wickedness appears to lie chiefly in the harm done by unauthorised vendors to the legally qualified druggist, whose trade is taken from him by the insatiable desire to have cheap drugs irrespective of their purity. Apart from this it is better that quacks should sell paregoric without opium than with too much.

Are Londoners going to make preparations for a visitation of cholera in the coming year, or trust that we can safely look for such immunity as we have experienced during the present year? There is only too much good ground for the cholera seed in the metropolis, if people would not so wilfully shut their eyes to the fact. It seems to be the hardest thing in the world to get rid of filth and pollution even when pestilence threatens.

Students of natural history have pointed out numerous instances of animals and birds adapting themselves to changed conditions of life. A somewhat interesting example of this is asserted to be taking place at the present time in Australia. The fleeces of the strains of English sheep imported into that continent are said to be growing decidedly lighter, owing to the fact that the heavy fleece needed for protection in our climate is not necessary under a warmer sky. The change is doubtless pleasant for, and duly appreciated by, the sheep, but not so pleasant to their owners, who look to the fleece for their profits.

